# Pattern of use of personal protective equipments and measures during application of pesticides by agricultural workers in a rural area of Ahmednagar district, India

**DOI:** 10.4103/0019-5278.58915

**Published:** 2009-12

**Authors:** Bhoopendra Singh, Mudit Kumar Gupta

**Affiliations:** Department of Forensic Medicine and Toxicology, Rural Medical College, Loni, Ahmednagar, Maharashtra, India

**Keywords:** Agricultural worker, personal protective equipment, pesticide application, protective measure

## Abstract

**Background:**

Pesticides, despite their known toxicity, are widely used in developing countries for agricultural purposes.

**Objectives:**

To find various patterns of hardware use for spraying of insecticides, prevalent storage practice adopted by the user, types of personal protective equipments used for the handling of chemicals; to detect dangerous practices and the extent to which safety norms being followed by the users during the application/treatments, and finally their knowledge concerning the risks of pesticides.

**Materials and Methods:**

The agriculture workers who had been involved in pesticide application for agricultural purpose were interviewed face-to-face to gain information on the following determinants of pesticide exposure: Types, treatment equipment, use of personal protection and safety measures during the application/treatments and knowledge of the risks of pesticide exposure.

**Results:**

Hundred workers, aged between 21 and 60 years old, were included. Pesticides were mostly applied with manual equipment using Knapsack (70%) and only 5% farmers were using Tractor-mounted sprayer. Workers frequently performed tasks involving additional exposure to pesticides (mixing chemicals, 66%, or washing equipment, 65%). Majority of the workers/applicators used no personal protection measures or used it defectively/partially. Most of the workers/respondents (77%) did not bother for safety and health risks of pesticide exposure.

**Conclusions:**

Workers involved in pesticide application use personal protection measures very poorly and defectively. Almost half of the applicators were not following right direction with respect to wind direction while spraying, thus it increase the risk of exposure. There is a clear need to develop specific training and prevention programs for these workers. The determinants of pesticide exposure in agricultural workers described in this study should be properly assessed in epidemiological studies of the health effects of pesticides on agricultural workers at national level.

## INTRODUCTION

Pesticide poisoning is a major global health problem, and it is more prevalent in countries like India. The harmful effects on human beings in the form of acute and chronic toxicity exposed to insecticides are well established. The incidence of pesticide poisoning is increasing according to the existing reports, and it is estimated that about 5 million people die every year as a result of intentional, accidental and occupational exposure worldwide.[1]

Furthermore, chronic exposure has been linked to other health problems, such as polyneuropathy, dermatitis, behavioural changes, and cancer as stated in a number of publications. The accidental chronic poisoning due to failure in adopting proper preventive measures during spraying/application is known, but only very few studies have been carried out in this direction. The great majority of farmers exposed to the insecticides are illiterate and are not aware of proper toxic effects and preventive measures. Absorption of insecticides through the skin, respiratory passages or by oral route may result in acute toxicity. Long-term exposure to the sub-toxic doses results in harmful effects on various systems viz. neurotoxicity, neuro-endocrinotoxicity, carcinogenicity, etc.

The objective of this study is to find out the patterns of uses of preventive measures for the safe use of insecticides.

It is observed from the results of the present study that the great majority of pesticide applicators were not using protective gears and measures. Considerable/significant numbers of the respondents were not following recommended safety measures.

## MATERIALS AND METHODS

Various methods of application of pesticides used by the agricultural workers from 100 houses of the selected village (Loni and Pravaranagar) of the district Ahmednagar were studied. The agricultural workers who had been involved in pesticide application for agricultural purpose were interviewed face-to-face after obtaining given consent to gain information on the patterns of hardware use for spraying of insecticides, prevalent storage practice adopted by the user, types of equipments used for the handling of chemicals, and the extent to which safety norms being followed by the users during the application/treatments and knowledge concerning the risks of pesticide exposure, and the results were recorded. Pre-designed standard performa was used to gather the information. The data was analyzed using SPSS - 10.

## RESULTS AND DISCUSSION

The patterns of hardware used for spraying of pesticides, prevalent storage practices adopted by users, and type of pesticide application equipment used for pesticide application were studied.

As a part of research project, survey of pesticide application pattern was conducted in the selected villages of Dist. Ahmednagar (Maharashtara) to learn more about the current safety gear used, pesticide application and disposal of empty pesticide container practices. The results of the survey based on the views of pesticide applicators are presented and discussed here:-

### Protective gears used

Majority of pesticide applicators were not using recommended protective gears. Only 23% of the respondents were in the recommended complete clothing while handling pesticide. Forty percent of pesticide applicators used to take bath and wash their clothes every day after spraying. Majority (93%) of the respondents were not using goggles and some of them (33%) were not using gloves during spraying. People of this area were conscious about inhalation exposure and 81% of the respondents were using cloth on face and 5% of the respondents were using dust mask during mixing/application of pesticide. Majorities (35%) were barefooted doing pesticide spraying, 33% applicators were using slippers and only 32% applicators were wearing shoes. Nobody was wearing gumboots [Table 1]. People were not using recommended protective gears either due to discomfort faced due to protective gears or due to ignorance. Similar findings were reported from the study conducted by Dogra *et al.*[2]

**Table 1:** Distribution of subject in respect of protective gears

Protective measures and gears	Participants (*n* = 100)
Clothing	
Complete	23
Partial	77
Ablution	
Cloth changing	60
bathing and wahsing cloth	40
Goggles	
User	07
Non user	93
Gloves	
User	67
Non user	33
Nose protection	
Gas mask	00
Dust mask	05
Cloth on face	81
None	14
Foot wear	
Gum boot	00
Shoes	32
Slipper	33
None	35

### Safety measures

Out of 100 respondents, majority (59) had red the instruction given in the pesticide safety manual and only 23% were not following the instructions given in the safety manual. Majority of the farmers did not bother about safety. It is observed that considerable proportions of the respondents (20%) were smoking, chewing tobacco or consuming other items during pesticide application, which is not acceptable in any case. Eighteen percent (18%) of the respondents were working alone while handling highly toxic pesticide. The findings of this study slightly differ with the findings of Dogra *et al.,*[2] based on the fact that the number of the respondents who were smoking, chewing tobacco or consuming other items during application were less as compared to the present study.

Many pesticide applicators (34%) did not follow right direction with respect to wind direction, which may increase exposure with pesticides, which in turn adversely affect application efficiency. The number of applicators who were not following right direction with respect to wind direction were more in the study of Dogra *et al.,*[2] as compared to the present study. About 50% of the respondents had checked their equipments for working/leakage before use. To check the leakage of equipments, most of the farmers were using soap, rubber tube, cloth etc. in exceptional cases, they were changing connecting pipes and most of the respondents were not doing regular servicing of equipments.

### Storage of pesticides

Farmers of these villages generally avoid storage of pesticides due to its easy availability and risk of health hazards if not stored properly. Fifty percent of the respondents had kept pesticide in original containers at field and the remaining respondents stored pesticides at home, which is not recommendable in terms of safety point of view.

### Method of application

Seventeen percent of the respondents were using Knapsack sprayer due to its easy availability and suitability for their crops in spraying. However, some farmers (5%) were using tractor-mounted sprayer and 34% were using manual sprayer. Majorities (44%) were using some other method (manual) for spraying the pesticide. In comparison with the findings of Dogra *et al.,*[2] it is observed that farmers in this area are more ignorant about the development of health hazards due to wrong application practice of pesticides, as 44% of farmers were using other methods (manual) for spraying the pesticides.

### Pesticide container disposal practices

The survey included questions mostly related to the management and disposal of left over pesticides and empty (used) pesticide container. Proper rinsing is necessary so that it may not contaminate the surrounding atmosphere and ground water. Most of the pesticide applicators (45%) sold the empty containers after rinsing and 34% disposed the containers without proper rinsing/washing which is not acceptable. Only 12% of the respondents were using empty containers for domestic purpose after well rinsing, which is also not a healthy practice. The remaining respondents left empty containers in the field after use, which is also not acceptable.

### Exposure to pesticides

This part of survey included questions related to make up of participants and their involvement in pesticide application. It is difficult to estimate the correct duration of pesticide exposure for each pesticide applicator because most of them are illiterate and they do not maintain the record of such practices. A thumb rule was adopted to estimate relative exposure to pesticides. The number of years of pesticide exposure and number of sprays done by each pesticide applicator per year were recorded.

It is evident from the analysis that majority (55%) of pesticide applicators was of the age range 21-40 years [Table 2]; out of these 100 respondents, 75 were spraying pesticide 10-30 times per year. Major proportions of the respondents (82%) were exposed to pesticides for 6-15 years. Therefore, it may be concluded that people of the age range 21-40 year were relatively highly exposed to pesticides.

**Table 2:** Age-wise distribution of participants (*N* = 100)

Age group	Number (%)
21-30	25 (25)
31-40	30 (30)
41-50	20 (20)
51-60	13 (13)
> 60	12 (12)

The dermal route is considered as the major route of exposure during the handling of pesticides.[3] Several methods are used for monitoring dermal exposure, such as washing or wiping, pseudo-skin methods, biological monitoring and video-imaging.[4] The ‘whole body’ technique[5] is a method to determine the potential toxicity and actual exposure by analyzing the active substances in the clothing preferably work clothing and underclothing, as well as inhalation monitoring and biological monitoring. The present study proposes to estimate the effectiveness of use of PPE by using the whole body technique.

Most of the studies showed that the use of personal protective equipments, changes in application procedures, packaging, mixing, and biological monitoring reduced the pesticide exposure under controlled conditions.[6] Cholinesterase monitoring and urinary metabolites can identify workers with a higher risk of overexposure. Most studies suggested that the interventions should be examined for their ability to reduce pesticide overexposure in actual working populations.[7–8]

Lander and Lings[8] found that cholinesterase enzyme (ChE) activity decreased as weekly spraying time increased among greenhouse applicators. They found that gloves were helpful in preventing ChE inhibition.[9] Putnam *et al.,* reported reduced pesticide exposure with the use of rubber gloves, but concluded that gloves did not completely eliminate exposure when working with vegetable crops treated with nitrofen.[10]

Some studies have showed that although most of the farmers are aware of the importance of use of protective measures when applying pesticides, there is no significant positive relationship between awareness and use of protective measures. The main reason for not using protective measures is discomfort.[11] These findings are corroborating with the findings of the present study.

## CONCLUSION

- Organophosphate group of pesticides are the most commonly used pesticides by the farmers in this area.
- Among the Organochlorine group of pesticides, the farmers commonly use the endosulfan.
- Majorities of pesticide applicators were not using protective gears.
- Considerable/significant numbers of the respondents were not following recommended safety measures.
- Almost half of the applicators were not following right direction with respect to wind direction, thus increasing the risk of exposure.

### Suggestions

Following factors were contributing to the risk of pesticide toxicity:

- Lack of suitable protective clothing for tropical climate.
- Poor knowledge and understanding of safe practices in pesticides usage.
- Farmers are not aware of exact pesticide concentration; sometimes, the concentration is excess.
- Poor maintenance facilities for spray equipment, thereby giving rise to hazardous contamination.
- Use of pesticide mixture in some cases.

Therefore, the following steps are recommended to reduce pesticide poisoning up to some extent:

1. Manufacturers of pesticide/pesticide application equipment must organize training programmes, by professionally trained people preferably agricultural engineers, for all persons related with pesticides (production, formulation, storage, transportation, application, monitoring etc.).
2. Ensure proper monitoring of pesticide consumption at state and national level so that its misuse can be restricted.
3. There should be facilities of periodical health check-up for pesticide applicators at least at Taluka/district level so that toxic hazards/chronic poisoning could be detected at initial/early stage and precautionary measures can be adopted.
4. There should be service/monitoring centres for spraying equipmenta at least at Taluka/District level so that faulty equipment can be repaired/replaced before it create health hazard to human being.
5. Further study should be conducted for suitable protective clothing/preventive gears subject to the terms and conditions of farmers of different parts of country.
6. There is need to carry out study to evaluate the risk of toxicity due to pesticides using control group.
